# Assessing Writing Motivation: a Systematic Review of K-5 Students' Self-Reports

**DOI:** 10.1007/s10648-023-09732-6

**Published:** 2023-02-23

**Authors:** Aline Alves-Wold, Bente Rigmor Walgermo, Erin McTigue, Per Henning Uppstad

**Affiliations:** Norwegian Centre for Reading Education and Research, University of Stavanger, Stavanger, Norway

**Keywords:** Writing, Motivation, Elementary education, Systematic review, Measures, Self-report

## Abstract

For attaining success in writing, motivation is essential. Crucially, instruction is dependent on knowing the student’s capabilities and inner drives. To date, research on writing has yet to establish a consistent framework for assessing writing motivation, and often fails to acknowledge students’ self-reports, rather favoring evaluations of students’ writing motivation made by others, such as teachers and researchers. This limbo state originates partly from a general skepticism towards the trustworthiness of elementary students’ self-reports. Nonetheless, the validity of such self-reports has been acknowledged in adjacent fields, such as reading. Aiming to establish a knowledge base from studies addressing students’ voices, the present study adopts the method of a systematic review and investigates how writing motivation has been assessed in empirical studies (1996–2020) through K-5 students’ self-reports. Of the 7047 studies identified through database search, 56 met the inclusion criteria and are examined in this review. Results indicate that (a) *storytelling* is the genre most used to operationalize writing in the investigations, (b) surveys and interview questions measuring students’ *attitude* towards writing are the most common type of self-report used, and (c) students’ voices are weighted differently across the studies. Findings suggest that future research should (1) work to counteract existing biases in writing tasks, (2) provide a rationale for their choice/design of measure of motivation, and (3) report clearly whose voices are being heard (e.g., students’, teachers’, or researchers’) and the appropriateness of this choice regarding study purpose, design, and findings.

**Supplementary Information:**

The online version contains supplementary material available at 10.1007/s10648-023-09732-6.

## Introduction

Successful instruction is dependent on knowing the students’ capabilities. As the Danish philosopher Kierkegaard stated: “If one is truly to succeed in leading a person to a specific place, one must first and foremost take care to find him where he is and begin there. This is the secret in the entire art of helping” (Kierkegaard, 1859/1998, p. 45). Accordingly, to gain insight into individuals’ inner states, it is essential to obtain their own perspectives; therefore, the students’ voices must be heard. Facing 20–30 students in a classroom, teachers have limited possibilities of tapping their students’ inner states through observation alone. Instead, teachers should aim at obtaining students’ own perspectives as a primary source.

In assessment theory, the search for the optimal source of information is a crucial issue for validity. However, in research on writing motivation, discussions of optimal sources for obtaining information on students’ motivation to write remain absent. For instance, recent reviews on writing motivation (Camacho et al., 2021; Ekholm et al., 2018), have analyzed and summarized findings regarding students’ motivations to write without clearly delineating whether these findings were based on teachers’ and researcher’s evaluations of the children’s inner states or formulated by the children themselves. Foregrounding the importance of listening to students’ own perspectives to advance our understanding of writing motivation, we acknowledge that, in this quest, valid instruments to capture their voices are needed. In the present review, to contribute with knowledge on current uses of students’ self-reports for assessing writing motivation, we analyze self-reports used in empirical studies, and provide an overview of how aspects of both writing and motivation were addressed in these self-reports, and how students’ voices were emphasized in these studies.

## Developing Writing Motivation in Early Elementary Settings

Before proceeding further, we must pause and address the consequences of learning to write in the early school grades. Hardly, a controversial point—there is disciplinary consensus that *proficiency* with the written word is essential for both school and life success, and early experiences with writing can predispose children to either seek out or avoid writing (NELP, 2008). Where the disciplinary consensus begins to falter is in the degree of recognizing the role of *motivation* in writing attainment. Like many current researchers (Boscolo & Gelati, 2018; Camacho et al., 2021; Ekholm et al., 2018; Klassen, 2002), we take the stance that developing writing skills requires much persistence, therefore writing research and instruction cannot only focus on *skills* but must also continually consider *motivation*. Specifically, to support young writers’ motivation, we need to communicate value about writing and gain knowledge about their envisioned goals, interests, and self-beliefs. Yet, only recently, questions regarding the role of motivation for writing development and how to promote it through classroom practices have begun to reach the center stage of writing research, which leads us back to the importance of finding out *where students are* and *meeting them there*. However, at this point, there is very little consensus on assessments of writing and of writing motivation in particular—especially for younger writers.

This overall lack of research about writing motivation in early stages of education is problematic when one considers the particular importance of initial writing experiences for motivation. In general, success builds beliefs in one’s efficacy, while failure undermines such self-beliefs (Zimmerman, 2000). These mechanisms are particularly evident in early phases of skill development where failure typically occurs before a sense of efficacy has been firmly established (Bandura, 1995). This implies that children in their first years in school have writer self-beliefs that are particularly malleable and dynamic (Unrau et al., 2018). Consequently, the first years in school represent both great opportunities and potential threats to writing development.

Additionally, a second problematic trend is that although children often arrive at school with *intrinsic motivation* to write, as formal instruction progresses, students tend to shift in orientation to *extrinsic motivation*—such as grades (Boscolo & Gelati, 2007). Instruction that was attuned to motivation (i.e., informed by assessments of motivation) would ideally maintain or strengthen intrinsic motivation. However, as Troia et al. (2013) remark, unlike in reading research, there is no systematic research to document how, how much, or why writing motivation may diminish over time. We argue that this lack of knowledge is indicative of the fact that writing motivation is rarely assessed in schools. For instance, recent large-scale studies in England (Dockrell et al., 2016), the USA (Brindle et al., 2016), and the Netherlands (Rietdijk et al., 2018) have worked to document the common instructional practices for writing in elementary grades, and despite cataloging a wide variety of practices and assessments, none of the studies documented efforts to assess students’ motivation.

Due to the limited research in writing motivation, we can consider the related field of reading research, for potential insight as to immediate needs and directions for writing motivation. For example, we now have meta-analyses that document bi-directional relationships between early reading skill and motivation (e.g., Toste et al., 2020), but this research is still to be expanded for writing before firm conclusions can be drawn. We also need to acknowledge that such advances in reading research have been obtained—to some extent—by the presence of validated models of assessment for elementary (e.g., MRQ, in Wigfield & Guthrie, 1997) and early childhood (e.g., SELM, McTigue et al., 2019).

Yet, recognizing that motivation is contextual (Troia et al., 2012), we cannot simply transpose knowledge from the domain of reading motivation to writing motivation. As early as first grade, attitudes towards writing form a unique construct compared to attitudes for reading (Graham et al., 2012a, 2012b). In fact, motivation to write may be even more important for literacy attainment than reading motivation because, simply put, writing is harder than reading because it is a production task demanding a complicated series of decisions and actions (Møller et al., 2022). As Bruning and Horn (2000, p. 26) aptly describe: “Students need to be motivated to enter, persist, and succeed in this ill-defined problem space we call writing.”

In line with such a domain-specific view of motivation, Troia et al., (2012, p. 7) have reviewed motivation research in the specific domain of writing and argue that *four broad components of motivation* have been identified: (1) *self-efficacy beliefs* (Bandura, 1986, 1994), (2) *goal orientations* (Dweck & Leggett, 1988; Elliott & Dweck, 1988; Harackiewicz et al., 2002), (3) *task interest* (Hidi, 1990; Hidi et al., 2002) and value (Eccles et al., 1983; Wigfield & Eccles, 2000), and (4) *outcome attributions* (Schunk, 1994; Weiner, 1986). In their review, a schema is also proposed, portraying the interrelationship between these four motivational components and associated constructs, such as domain self-concept and task utility (Troia et al., 2012, p. 11). However, although this schema portrays how motivation constructs are interrelated, Troia et al., (2012, p. 11) point out that some links, such as the causal pathways between self-efficacy, interest, and value, are still unclear. To help untangle these causal connections, those authors then invite researchers to test these connections by combining different research methods, such as classroom observation and students’ self-reports (p. 18).

## Quality of Self-Reports Measuring Writing Motivation

Over the past decade, there has been an enlarged focus on the documentation of the quality of assessments overall in education (e.g., Arnesen et al., 2019; Evers et al., 2013), but the positive effects of this focus have not appreciably impacted the assessment of writing motivation. Furthermore, being that motivation is an internal state of mind, assessments must include self-reports and not only rely on others’ (e.g., teachers or parents) interpretations of behavior in order to most validly capture motivation. Yet, among existing approaches, there is a large variation regarding the extent to which students’ self-reports are considered. In addition, as our focus includes writing in K-5 grades, there are additional challenges to consider when measuring young children’s motivation (McTigue et al., 2019), as they may not be able to communicate their thoughts and feelings as well as older learners. Therefore, attempts at measurement are often compared with the ambition of hitting a moving target.

These circumstances touch upon central validity issues, and in particular *construct validity*, often referred to as the core of validity, concerned with measuring the construct in question as accurately as possible. Discussions on validity today, however, more commonly target stakeholders’ (here both the researchers’ and the teachers’) interpretations and use of test scores (Kane, 2006, 2017; Lane et al., 2016, p. xv) in parallel with more common aspects of validity expressed in psychometric terms and standards (e.g., reliability). This practice embraces a unitary view of validity—in which no aspect of the validation process in principle is superior to the other. However, elaborating on the unitary view of validity, Kane (2013) states that potential scenarios for interpretation and use of test scores should be highlighted systematically even before the development of a test starts. That is, considering validity only during the interpretation phase is too late. In other words, valid test development starts by considering potential scenarios for the interpretation, using test scores, and evaluating limitations and weaknesses that may threaten valid interpretation and use of the test.

In the present study, the focus on students’ voices acknowledges the appropriateness of argument-based validity in important ways. First, it values students’ own perspectives on their motivational states as mostly relevant to both the use and interpretation of these measures. Second, it emphasizes the measurement itself, meaning that it must be understandable to its users, including teachers, students, parents, and policymakers.

## Scope of the Present Review

Although different literature reviews about students’ writing motivation have recently been published (Camacho et al., 2021; Klassen, 2002; Troia et al., 2012), to our knowledge, no study focused on the measures used to capture students’ voices on this matter. Although Ekholm et al. (2018) address conceptualization and measurement issues in their review, they focus exclusively on writing attitudes, whereas we approach motivation more broadly. In addition, we bring attention to the necessity for children’s voices to be given primacy and not their behaviors (which are interpreted through others) or the voices of adults. Indeed, as a crucial dimension of the United Nations Convention on the Rights of the Child (UNCRC), it is stated that if we want to know what is actually in the interest of the child, it is logical to listen to him or her (UNCRC, 1989; article 12:1). This emphasis on listening to the students’ own perspectives is a radical position assuming that assessment of motivation to write should be built first and foremost on the students’ voices—the optimal and primary source of information of their inner motivational drives.

First, we address the phrase “motivation to write,” acknowledging the impossibility to focus on *motivation* without also addressing what these drives are *directed towards*, namely *writing*. Walgermo et al. (2018) name the relation between motivation and the *target of motivation* in ecological terms as being symbiotic, and Walgermo and Uppstad (in press) state that “Unlike reading or writing skills, which are often studied for their own sake, motivation is a potential that is most typically investigated in relation to other potentials, like reading and writing.” In line with this remark, we first investigate the types of writing tasks addressed in self-reports measuring students’ motivation to write.

Next, as remarked by Camacho et al., (2021, p. 234), an array of motivation-related constructs has often been presented in writing research without being explicitly defined, which, according to the authors, leads to “conceptualization issues and terminological overlaps.” However, we argue that not clearly defining motivational constructs in a study has consequences, not only for the conceptualization of these constructs but also for their measurement. That is, if a construct such as ‘self-concept’ is not clearly defined in a study, how can it be accurately measured to portray the students’ voices? Thus, in the present review, we seek to identify what motivation constructs are measured and how they are operationalized in studies investigating early elementary students’ motivation to write.

Finally, given the claimed inattention of what is the primary source of information when investigating students’ motivation to write, there would likely be an expectation of a large variance in how students’ voices are actually valued and weighted across studies, so we investigate how much primacy students’ voices are given in the identified studies.

Within this scope, we wish to identify strengths and weaknesses in current measurement to inform future research, and these areas of timely inquiry are addressed in the following three research questions:

1. What types of writing tasks are addressed in self-reports measuring students’ motivation to write?
2. What motivation constructs are measured and how are they operationalized?
3. What emphasis is given to students’ voices in the studies?

## Method

The present study has adopted the methodology of a systematic review (Gough et al., 2012), which is usually conducted through five steps: (1) framing the research question(s) that will guide the review (as presented in the previous section), (2) identifying relevant work through systematic literature search and pre-defined selection criteria, (3) assessing the quality of the studies identified, (4) summarizing the evidence from the selected studies, and (5) discussing the findings (Khan et al., 2003). Where applicable, the present review follows guidelines from the Preferred Reporting Items for Systematic Review and Meta-Analyses (PRISMA) (Moher et al., 2009).

### Identifying Studies

#### Rationale for the Review Timeframe

The present review includes studies that were published between January 1, 1996 and April 1, 2020. The year of 1996 has been chosen as the starting point of the review for two reasons. First, according to Hidi and Boscolo (2008, p. 144), the impressive body of research on motivation that advanced during the 1980s only impacted writing research much later, when writing researchers demonstrated that writing is a complex task requiring not only the coordination and development of cognitive skills, but also *affective components.* Hayes’ revised framework, published in 1996, reflects this new conceptualization of writing, where affective components are given a much more prominent role. Second, as proposed by Alexander and Fox (2004, p. 50), the period from 1996 onwards is seen as the “Era of Engaged Learning,” a period characterized by a shift in the way the literacy community perceived and investigated learners and learning, and in which researchers began to look at motivational components, such as goals and interests, as critical factors for learning development. Thus, given the significance of the year 1996 for writing motivation research in educational settings, it has been chosen as the starting point for the current review. This being said, any cut-off dates are likely to be more or less indicative rather than rigid, as the educational research literature tend to have few clear-cut joints. We acknowledge that there were studies on writing motivation before 1996—as will be the case for any time frames set—but the chosen year is here supported by an indication that something culturally changed, which marked a “key turning point” in writing motivation research.

#### Systematic Literature Search

**Fig. 1:**
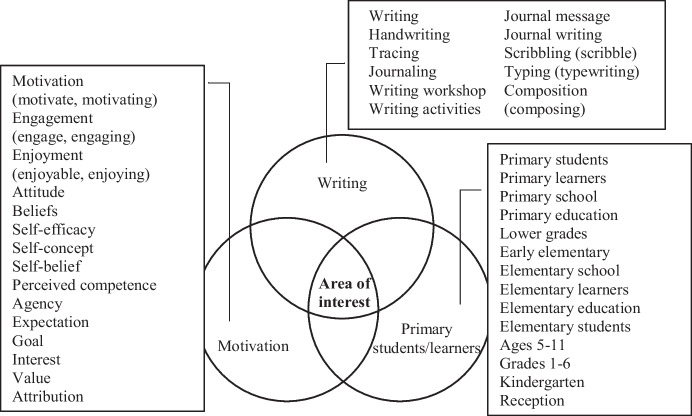
Diagram of search terms clusters

A thorough search of the literature was conducted using four different databases: ERIC, Academic Search Premier, PsycINFO (1806–present), and Web of Science. The scope of this review—as formulated in the RQs above—addresses three main topics, namely: *writing*, *motivation*, and *early elementary education* (including K-5 grades). From each of these three topics, related search terms were added, providing a total of 49 search terms (see Fig. 1). Figure 1 portrays the area of interest for the present study in visually representing the intercept of the three main topics—with related terms—addressed in the present review.

The initial literature search returned 12,839 records. Thereafter, depending on their availability within each database, limiters matching some of the inclusion criteria discussed below were applied, and a total of 7047 studies were retrieved for screening. These studies were then exported to EPPI-Reviewer, a software tool for research synthesis, where 1252 duplicates were removed. This resulted in a total of 5795 studies that moved to the screening stage of this review, as summarized in Table 1.

**Table 1:** Total of records retrieved for screening

Database	Initial search	Limiters applied	Records retrieved for screening
PsycINFO (1806–present)	1244	Peer-reviewed, 1996–2020	551
ERIC	7419	Peer-reviewed, 1996–2020	2766
Academic Search Premier	1882	Scholarly, 1996–2020	1542
Web of Science	2294	1996–2020	2188
	**12**,**839**		**7047**
		Duplicates excluded	1252
		Total for screening	**5795**

#### Selection Criteria

In EPPI-Reviewer, the remaining 5795 studies were screened manually, first on title and abstract, and then on full text. Following the lead of Miller et al., (2018, p. 89), the inclusionary criteria for selecting studies in both phases were divided into four categories as shown below:

- Publication: articles were written in English and published between January 1996 and April 2020
- Research: studies were empirical and peer-reviewed
- Topic: studies focused on students’ writing motivation
- Participants: studies focused on K-5 students in mainstream classrooms (studies that focused solely on second language learners or students with special needs were excluded)

In phase 1 of the screening process, 5795 studies were screened on title and abstract and based on these criteria, 5434 studies were excluded in this phase. Then in phase 2, 361 studies were screened on full text; and based on the same criteria, 267 studies were excluded in this phase, which resulted in a total of 94 studies. Similarly to Hakimi et al. (2021), these stages were carried out by the first author, but the other authors were available to discuss abstracts and titles that were ambiguous or presented uncertainty.

As the process of a systematic literature review (SLR) is recursive, it allows researchers to adjust the procedures to maintain a focus on the research questions. At this stage, we noticed that in some studies, writing motivation was not directly investigated; it was rather used as a post hoc explanation of why students behaved in specific ways. Consequently, to ensure that the selected studies were directly focused on writing motivation, and the students’ perspectives were included, a third screening phase was added, incorporating the following two eligibility criteria: (1) the study has to include at least one research question about writing motivation (either explicitly stated in question format, or implicitly in terms of goals and purposes of the study), and (2) the study has to include at least one type of *student self-report* on writing motivation (e.g., survey, questionnaire, interview).

Articles meeting these criteria, but solely focusing on instrument development and validity were excluded. In this work, we are synthesizing research regarding students’ motivation in the classroom (i.e., the focus of our research questions is on classroom research), and validation research is focused more narrowly on establishing credibility of a measure which is fundamentally different. Student’s motivation will be measured in such work but only for the purpose of establishing reliability and validity of scales. In addition, to avoid overemphasis of particular studies, in cases where multiple articles reported on the same data, the latest version of the study was kept, and previous duplicate studies were excluded (e.g., Li & Chu, 2018).

In phase 3 of the screening process, 94 studies were screened on full text. Based on the eligibility criteria for this phase, 44 studies were excluded, which resulted in a total of 50 studies to be included in the corpus for this review. To ensure the reliability of this screening step, a random selection of 27% of the articles (i.e., 26 out of 94 articles) was double coded by the second author for eligibility. Interrater agreement was 92% (24 out of 26 articles), and 100% after discussion.

Finally, three different hand-search procedures were conducted. First, we conducted *backward snowballing*, where we hand-searched the reference lists of all 50 studies included in the review and identified two additional studies. Of these, only one could be retrieved, which resulted in 51 studies. We tried to retrieve the other study by contacting the authors who conducted the study, the authors who referenced the study, and the journal who published the study, but to no avail. Second, in trying to identify newer studies, we conducted *forward snowballing*. For this search step, we used both Scopus and Google Scholar to identify all the papers who had cited the 51 included studies in our review, and screened these references as described by Wohlin (2014). This search step led to the inclusion of two additional studies, totaling 53 studies. Third, we hand-searched the reference lists of six relevant reviews/meta-analyses (Camacho et al., 2021; Ekholm et al., 2018; Graham & Perin, 2007; Graham et al., 2022, 2012a; 2012b; Troia et al., 2012) and identified three additional studies that met our criteria, which resulted in a total of 56 articles included in the corpus for the present review. Backward and forward snowballing was again conducted for these additional studies, but new studies meeting our inclusion criteria were not identified. Figure S1 in the supplementary material provides numerical totals for each phase of the screening process.

### Assessing the Quality of the Studies

Before synthesizing results in a systematic review, it is common to assess the quality of these studies and exclude from review those that do not meet pre-defined quality criteria. However, according to Pawson et al., (2005, p. 30), “to synthesize *is* to make sense of the different contributions”, thus, the quality of studies should be “*established in synthesis* and not as a preliminary pre-qualification exercise” (originally italicized). In the present review, the quality of the studies is therefore assessed as part of the synthesis, rather than used as a screening step for excluding studies from the review.

To assess the quality of the studies, a methodological quality score (MQS), adapted from Goodson et al., (2006, p. 313) and Miller et al., (2018, p. 90), was used to evaluate each study. Table 2 shows the six criteria used in the assessment, and their full score leads to a maximum of 14 points per study. Since ternary categories that are divided among *yes*, *partially*, and *no* award 2 points for *yes* and 0 point for *no*, the same scores of 2 for *yes* and 0 for *no* is also used for binary categories to maintain the balance in the scores.

**Table 2:** Criteria for assessing the studies’ methodological quality

Methodological characteristic	Scoring options (Maximum total score = 14 points)	Distribution of characteristics among 56 reviewed studies	
		Frequency (*n*)	Percent (%)
Explicates theory and/or previous research in a way that builds the formulation of the posed question(s)/purpose(s)/objective(s) that can be investigated empirically	Yes = 2 points	37	66
	Partially = 1 point	19	34
	No = 0 point	0	0
Research method	Quantitative or qualitative methods = 1 point	32	57
	Mixed methods = 2 points	24	43
Sample size	Narrow sample = 1 point	7	13
	Small sample (< 100) = 2 points	27	48
	Medium sample (> 100 and < 300) = 3 points	17	30
	Large sample (> 300) = 4 points	5	9
Refers to/uses relevant theory to justify choice/design of motivation measure	Explicitly = 2 points	19	34
	Implicitly = 1 point	27	48
	Does not refer to relevant theory = 0 point	10	18
Characteristics/evidence of validity, reliability, credibility, and/or trustworthiness are addressed and reported	Reported = 2 points	51	91
	Not reported = 0 point	5	9
Findings and conclusions are legitimate or consistent with data collected	Yes = 2 points	51	91
	No = 0 point	5	9

### Coding and Analysis of Eligible Studies

Following the lead of Reed et al. (2014), the coding process was carried out through three stages. In the first stage, a coding sheet was developed and refined by the four authors. In the second stage, the first author coded all studies included in the corpus (*n* = *56*), and in the last stage, the second author double coded all 56 studies and discrepancies were resolved.

In stage 1 of the coding process, based on our research questions, all four authors met in a conference call to develop a spreadsheet divided into predefined categories (e.g., number of participants, type of self-report, motivation construct investigated, type of writing task) where the 56 included studies could be coded deductively. After initial categories were agreed upon, the first author coded five of the studies and met again with the other three authors to refine the coding scheme. The final version of the spreadsheet was organized into four main categories: (1) characteristics of the studies, (2) quality of the studies, (3) measures of writing motivation, and (4) factors affecting writing motivation. In the current review, we present results regarding the first three categories (for a detailed discussion of factors affecting writing motivation, see Alves-Wold et al.–in review).

During the second stage of the coding process, the first author coded all studies included in the review (*n* = 56), and all authors were available to discuss peculiarities of the studies and particularities of the coding. Finally, in the last stage, the second author double coded all 56 studies, and any discrepancies between the two researchers’ scores were resolved through a second review, discussion of discrepancies, and a finalized consensus. Throughout the process, in addition to informal meetings among the authors, all four authors met at least monthly on a conference call to discuss particularities of the study and make consensual decisions regarding each stage of the review process.

#### Characteristics of the Studies

Studies were coded for the following eight characteristics: name of scientific journal, year of publication, country where the study was carried out, number of participants, participants’ grade-level(s), research method, whether the study was an intervention, and a summary of the main findings (see Table S1 in the Supplementary Material for an overview).

#### Quality of the Studies

Notes were taken regarding the quality of the studies and scores were assigned for each study according to the six categories described in Table 2.

#### Measures

We coded the studies for type of writing task, type of student self-report, and details about the measures used in each study, such as if the measure was administered in a group or individually, or if images were used for support. In addition, we coded for type of triangulation data for studies that combined student self-report with other types of data.

## Results and Discussion

In this section, we synthesize results and findings related to the research questions guiding this review: (1) What types of writing tasks are addressed in self-reports measuring students’ motivation to write? (2) What motivation constructs are measured and how are they operationalized? (3) What emphasis is given to students’ voices in the studies? Before presenting results related to these, we describe general characteristics of the studies, followed by an overview of their quality.

### Characteristics of the Studies

#### Period

There is a significant growth in the number of studies published on writing motivation in early elementary education, as almost 70% (*n* = 38) of the 56 reviewed studies were published in the last decade, rather than between 1996 and 2009 (*n* = 18).

#### Place

Almost 60% of the studies were conducted in North America, with the USA contributing the most: USA (*n* = 28), Canada (*n* = 4), and Mexico (*n* = 1). Asia was the second most represented continent with 28% of the studies: Turkey (*n* = 9), China (*n* = 2), Singapore (*n* = 2), Indonesia, Jordan, and Taiwan (all *n* = 1). Europe contributed with 11% of the studies: Finland (*n* = 2), Cyprus, Italy, Portugal, and Sweden (all *n* = 1). Finally, Oceania contributed only one study originating from Australia. Consistent with the review conducted by Camacho et al. (2021), the present review did not identify any studies from Africa nor South America. Given that one of the inclusionary criteria for this review requires that studies need to be written in English, it is not surprising that more than 50% of the studies are from English-speaking countries; however, none of the studies originated from the UK.

#### Publication

The studies were published in 44 different peer-reviewed journals, with the most represented journals being *Early Childhood Education Journal* (*n* = 4), *Reading Psychology*, *Reading & Writing Quarterly* (both *n* = 3), *Education*, *Elementary School Journal*, *Reading Improvement*, *Reading Horizons*, and *International Electronic Journal of Elementary Education* (*all n* = 2).

#### Participants

Grade levels were investigated individually in 70% of the studies (*n* = 39). Most of these (*n* = 28) focused on the upper grade levels: 5th (*n* = 13), 4th (*n* = 9), and 3rd (*n* = 6), whereas only less than a third (*n* = 11) of these studies focused on the lower levels: Kindergarten (*n* = 6), 1st (*n* = 2), and 2nd (*n* = 3). More than one grade level was investigated in 30% of the studies (*n* = 17). Most of these studies (*n* = 12) focused on grade pairs, with the most common combinations being 2nd and 3rd (*n* = 3) and 4th and 5th (*n* = 3). Only one study included participants from all six grades.

### Quality of the Studies

MQS values were awarded to each study, as described in Table 2. Scores ranged from 5 to 13 points (maximum possible = 14), and the mean, median, and mode values were very similar to each other (mean = 10.23, median = 11, and mode = 11). Almost 75% of the studies received a score higher than 70% of the MQS, and only two studies received a score that was lower than 50%. Table 2 shows the frequency distributions for each category of the MQS, and additional comments regarding these categories are presented below.

#### Research Method

Approximately 45% of the studies (*n* = 25) used a mixed-methods design, instead of exclusively quantitative (*n* = 18) or qualitative methods (*n* = 13). It is logical that researchers triangulate multiple types of data sources when dealing with complex affective constructs or measuring both writing skills and motivation constructs.

#### Sample Size

The number of participants included in each study varied significantly. Sample sizes spanned from qualitative investigations of one (Leroy, 2000) or two students (Abbott, 2000; Andrzejczak et al., 2005; Perry et al., 2003) to a quantitative investigation of the writing disposition of 2315 fourth and fifth graders (Unal, 2010). One of the studies did not specify the exact number of participants (Lee & Enciso, 2017), but according to our categories (narrow, small, medium, and large), we coded this study as investigating a large sample, as it included a sample of 29 classrooms.

As some of the studies investigating multiple grade levels provided only the total number of participants, we could not differentiate the total of participants per grade level. However, a total of approximately 8000 participants are investigated in the reviewed studies.

#### Theoretical Foundation of Studies and Rationale for Design of Motivation Measure

Most studies (*n* = 37) presented relevant theory and previous research addressing both writing and motivation. Theories and models cited by authors include self-determination theory (e.g., Ryan & Deci, 2000), social cognitive theory (e.g., Bandura, 1986, 1997), social constructivist theory (e.g., Vygotsky, 1978), model of social self-interaction (e.g., Schunk, 1999), attribution theory (e.g., Weiner, 1986), and self-theories (e.g., Dweck & Master, 2009). Authors also cited relevant literature to address writing skills (e.g., Hayes, 1996) and to define motivation constructs (e.g., self-efficacy: Bandura, 1977). Nevertheless, more than 30% of the reviewed studies (*n* = 19) tend to present theory and research that focus mainly on writing but lack relevant references to motivation theory and/or research. That is, even though these studies include research questions that investigate motivational constructs, relevant motivation theory is not used to explain what these constructs entail. In addition, in studies where both writing and reading were investigated, authors tend to refer mostly to reading research.

Only a third of the studies (*n* = 19) explicitly referred to motivation theory in their methods section to explain the rationale for the design of the chosen motivation measure. Some referred to motivation theory to explain the choice of previously used measures, as Nicolaidou (2012) who argues that the Writer Self-Perception Scale was chosen because “it was grounded on Bandura's (1997) theory of perceived self-efficacy.” Whereas others referred to motivation theory to justify the content of the measure developed for the study, as Liao et al. (2018) who explained that “the content of the WIQ [Writing Interest Questionnaire] was developed based on the four‐phase model of interest development (Hidi & Renninger, 2006; Schraw & Lehman, 2001).”

Nevertheless, approximately half of the studies (*n* = 27) only referred to motivation theory in the theory section of their articles, without explicitly linking the significance of the presented theory for the design of their motivational measures. In addition, ten of the studies did not refer to motivation theory to justify their choice of motivation measure, neither in the theory nor the methods sections of their articles.

Given that motivation is a multi-dimensional and dynamic construct, not clearly defining which constructs that are being investigated and how they are being operationalized in the chosen instruments might have consequences for the validity of the measure, as it becomes unclear which motivational components that are being measured and how. In other words, theoretical clarity can provide a needed link between a complex construct (i.e., motivation) and the measurement of that construct. To promote standardization in the definition of motivation constructs, Camacho et al., (2021, p. 224) provide a list of definitions that can be helpful for researchers to bring clarity to their investigations of motivation constructs.

#### Validity, Reliability, Credibility, and/or Trustworthiness

Authors addressed issues related to the trustworthiness of the studies in 91% of the cases (*n* = 51); however, the quality of the evidence reported by the authors varied among the studies. For instance, whereas some authors gave detailed accounts of the methods used to ensure the integrity of their investigations, others briefly addressed these issues under the Limitations section of their articles.

Out of 36 studies that included quantitative measures of motivation, such as scales, 22 reported evidence of reliability, and 14 of validity. In most of the studies, Cronbach’s alpha internal consistency coefficient was reported as evidence of reliability, and confirmatory factor analysis was used for reporting evidence of validity. In four of the studies, authors referred instead to previous validation studies of the measures used, which might be insufficient, as although an instrument is reliable/valid for one sample, it may not be for another.

Out of 38 studies that included qualitative measures of motivation, such as interviews and open-ended questions, 35 provided evidence of credibility and trustworthiness. Most of the studies provided detailed information about the context, the participants, and the procedures used for collecting, coding, and analyzing the data.

It is important to notice that the number of studies employing quantitative and qualitative measures discussed here should not be totaled, as these methods are combined in mixed-methods studies.

#### Legitimacy of Findings and Conclusions

In 91% of the studies (*n* = 51), findings and conclusions showed consistency with the data collected. However, in 9% of the studies (*n* = 5), part of the conclusions was not clearly linked to the results. This discrepancy was mainly due to a lack of clear distinctions regarding the sources of the results, which impacts the validity of the findings. For instance, in some of the studies including responses from both teachers and students, it was not always clear which group had uttered the responses discussed. When conclusions are presented, it is therefore difficult to ascertain how much they represent the students’ voices.

### What Types of Writing Tasks Are Addressed in Self-Reports Measuring Students’ Motivation to Write?

Given that the focus of our work is on motivation to *write*, researchers cannot consider this without operationalizing writing, so we start with how writing is addressed in the studies.

#### Some Studies Directly Measure Writing Skill and Others Do Not

Whereas all studies are measuring aspects of motivation, only a subset of studies also directly investigate writing skill (33 of the 56). In the studies that directly consider writing skills, measurements of writing quality were used, and these included teacher-reported evaluations (e.g., Perry, 1998; Zumbrunn et al., 2019), researcher judgements (e.g., Hall et al., 2017; Teague et al., 2010), and students’ self-assessments (e.g., Bradford et al., 2016; Nicolaidou, 2012). Quality levels were most commonly judged by the use of rubrics that set standards for *skill-related aspects of writing*, such as text organization (e.g., Boyacı & Güner, 2018), ideation (e.g., Schrodt et al., 2019), spelling accuracy (e.g., Jones et al., 2016), length of composition (e.g., Liao et al., 2018), and audience-awareness (e.g., Gallini & Zhang, 1997). Typically, multiple aspects of writing are investigated in synchrony (e.g., a writing sample that is analyzed from multiple lenses), and even seemingly simple writing tasks like spelling require the judgement of multiple aspects, like handwriting legibility, directionality (words are written from left to right), and spelling accuracy.

Although writing skill measures are not the primary focus of the present review, we note the value of these quality assessments in the studies’ investigations of students’ writing motivation. For instance, by measuring students’ writing quality, researchers are able to compare motivation levels between high- and low-achieving students (e.g., Perry et al., 2003), check how calibrated students’ self-efficacy beliefs are in comparison to their actual performance (e.g., Kim & Lorsbach, 2005), or investigate if changes in performance affect motivation levels (e.g., Li & Chu, 2018).

#### Types of Writing Tasks Addressed in the Studies

Various motivation constructs, to be discussed in the next section, were measured in the reviewed studies either in relation to writing as a *general task* (e.g., “what makes you a good writer?” from Kim & Lorsbach, 2005) or as *specific tasks* (e.g., writing a story, revising a text). Of the various types of writing tasks used in the studies, narrative/story writing (*n* = 17) was clearly the most common genre investigated. Studies also included genres such as expository (*n* = 5), informational/explanatory (*n* = 2), and descriptive writing (*n* = 1). However, it is difficult to give a precise number of the specific types of tasks investigated in the studies, as researchers used very different nomenclatures and categorizations of writing tasks. For instance, some authors used specific terms such as “letter writing” (e.g., Chohan, 2011) in their description of the investigated tasks, whereas others used more general terms such as “authentic writing tasks” (e.g., Boyacı & Güner, 2018), or “various writing activities” (e.g., Paquette, 2008) to denote them. Other types of writing tasks included, for example, spelling activities (Jones et al., 2016), linguistic games (Boscolo et al., 2012), poetry writing (Andrzejczak et al., 2005), collaborative writing (Li & Chu, 2018), and high- and low-challenge writing tasks (Miller & Meece, 1999).

In 24 of the studies, researchers indicated whether writing tasks or surveys were performed on paper (*n* = 12), digitally (*n* = 6), or both (*n* = 6); however, in 32 of the studies, the technology used was not specified.

Writing tasks were also investigated in intervention studies (*n* = 33) measuring the students’ levels of motivation in relation to specific teaching practices (*n* = 25), such as using artwork as a pre-writing activity (Andrzejczak et al., 2005), participating in writing workshops (Hertz & Heydenberk, 1997, Pollington et al., 2001), or through a drama-based program (Lee & Enciso, 2017). In eight of these studies, motivation was also investigated in relation to the use of different digital tools such as mobile apps (Kanala et al., 2013; Sessions et al., 2016) and online blogging (Nair et al., 2013). In one study, researchers measured motivation to write before and after a common teaching practice, that is, instruction that prepares students for a state-mandated writing exam (Tunks, 2010).

#### Items and Responses

Most often, students were asked to answer questions about their writing motivation before and after executing writing tasks (e.g., Babayigit, 2019; Beck & Fetherston, 2003; Boscolo et al., 2012; Hier & Mahony, 2018; Ihmeideh, 2015; Liao et al., 2018), or only after working with them (e.g., Jones et al., 2016; Kim & Lorsbach, 2005; Miller & Meece, 1997, 1999; Wilson & Trainin, 2007). However, students also answered questions about writing motivation without being asked to perform specific writing activities (e.g., Hall & Axelrod, 2014; Mata, 2011; Merisuo-Storm, 2006; Unal, 2010; Zumbrunn et al., 2019). In addition, students were asked to answer questions about writing tasks that differed from those they were asked to execute. For instance, in Akyol and Aktas (2018), students were asked to write a *story*, but their survey included questions about *narrative*, *expository*, and *general* writing. Table 3 provides some examples of how writing was operationalized as either a general or specific task in both open- and close-ended questions.

**Table 3:** Examples of how writing was operationalized as either a general or specific task in items and responses

Writing operationalization		Open-ended			Close-ended	
		Sample item	Sample response		Sample item	Sample response
General task	Erdogan & Erdogan, (2013)	“Writing is like… because…”	“Writing is like a book, because like books my writings give other people information.”	Akyol and Aktas (2018)–Adapted from Codling and Gambrel (1997)	Knowing how to write well is …	- Not important - Kind of important - Important - Very important
Specific task	Bradford et al. (2016)	Upon completion of the second posttest, students wrote a final reflection essay responding to the prompt “In your opinion, is it best to use a rubric when writing or not? Why?”	- Assists in remembering to do all the things and check work - Helps you get 4 stars (achieve all the requirements)	Nair et al. (2013)	I think learning how to blog is important	SA = strongly agree; A = agree; N = neutral; D = disagree; SD = strongly disagree

#### Does the Type of Writing Addressed Matter?

As shown, the results indicate a split distribution regarding general or more specific writing tasks. Interestingly, when specific writing tasks are targeted in measures of motivation to write, storytelling/narrative writing is by far the most common genre investigated. However, although story writing is traditional in school, this choice is somewhat curious for motivation research when students are given the opportunity to rate their motivation for other genres. For instance, when given the opportunity to explore and produce their own informational texts, students’ confidence and interest in this type of text increased (Hall et al., 2017). This difference in the occurrence of writing genres addressed in the studies, combined with findings showing that choice of task will have an influence on motivation levels (Alves-Wold et al.–in review), suggest a present bias concerning wider aspects of writing. From an assessment perspective, this bias may represent construct under-representation (Cook et al., 1979), a challenge that should be addressed when taking the first steps towards a coherent framework for measuring motivation to write.

A question that needs further research is whether this overweight of storytelling tasks in measures of motivation to write is mirroring either (a) researchers’ oversimplifications of writing, where storytelling represents writing in general, or (b) storytelling is a task overrepresented in classroom practices. Findings from the present study indicate that when motivation to write is measured, often a too narrow approach to writing is taken. This means that in order to develop valid self-report instruments for writing motivation, we need measures to target writing tasks that reflect a wider variety of classroom practices.

### What Motivation Constructs Are Measured and How Are they Operationalized?

#### Motivation Constructs

Most of the studies (*n* = 36) investigated one motivational construct, but in 20 studies, two or more motivational constructs were investigated simultaneously. Following the authors’ terminology in each study, a total of 32 motivational constructs were identified. In Table 4, to systematize the variety of constructs identified in the studies, we categorized these constructs according to *theorized components* of motivation and *associated constructs*, as described in the work of Troia et al. (2012). Column 1 provides frequency numbers for each identified construct, and column 1 provides total numbers for the identified constructs in each category. Frequency numbers for associated constructs show that *attitude* was clearly investigated most often (*n* = 20), followed by *self-efficacy* (*n* = 11), and *motivation* (*n* = 9). These frequency numbers also correspond with total numbers for each category, showing that the theorized component of *interest and value* was investigated most often (*n* = 39), followed by *self-beliefs* (*n* = 28), and *motivation* (*n* = 11).

**Table 4:** Sorting of motivation constructs

Theorized components	Associated constructs
Motivation (*n* = 11)	Motivation (*n* = 9), intrinsic motivation (*n* = 1), and achievement motivation (*n* = 1)
Self-beliefs^a^ (*n* = 28)	Self-efficacy (*n* = 11), self-concept (*n* = 3), perceptions of themselves as writers (*n* = 4), self-perception (*n* = 2), self-perception of competence (*n* = 1), confidence (*n* = 1), anxiety (*n* = 1), performance expectancies (*n* = 1), beliefs (*n* = 1), writer identity (*n* = 1), perceived competence (*n* = 1), outcome expectation (*n* = 1)
Goal-orientations (*n* = 2)	Goal orientations (*n* = 1), cognitive engagement (*n* = 1)
Interest and value (*n* = 39)	Attitude (*n* = 20), interest (*n* = 5), value (*n* = 3), enjoyment (*n* = 3), task value (*n* = 2), perception/belief about writing (*n* = 2), liking (*n* = 1), writing disposition (*n* = 1), aversion (*n* = 1), perception of teacher writing enjoyment (*n* = 1)
Outcome attributions (*n* = 5)	Effort (*n* = 1), control (*n* = 1), support (*n* = 1), growth mindset (*n* = 1), attributions (*n* = 1)

^a^Given that *self-efficacy beliefs* are task-oriented, we use the broader cover term *self-beliefs* to represent this broad component of motivation and its associated constructs

#### Types of Self-Reports

Self-reports were used 83 times in the 56 reviewed studies to measure the abovementioned motivational constructs. Although authors have used different nomenclature, the types of self-reports fall into three main categories: (1) interviews, (2) surveys and questionnaires, and (3) alternative written responses. *Student interviews* were used 32 times and included discussion sessions, student–teacher conferences, portfolio-based conferences, and individual interviews where students answered orally a questionnaire that included ratings from 1 to 10, and also open-ended questions where they could give reasons for their ratings (Miller & Meece, 1999). *Surveys and questionnaires* were used 46 times, and it is noteworthy that often the terms *surveys and questionnaires* were used interchangeably. In the reviewed studies, both surveys and questionnaires were often described as a set of written questions/statements (items) where students could indicate their responses by choosing one of the options in a Likert scale. In ten of these studies, surveys also included open-ended questions. Finally, *alternative written responses* were investigated five times, through qualitative analyses of the narrative portion of students’ feedback on exit slips (Truax, 2018), the students’ final reflection essay on the use of rubrics (Bradford et al., 2016), the students’ drawings and written description of a recent writing experience (Zumbrunn et al., 2017), and the students’ completion of the metaphorical sentence “Writing is like… because…” (Erdoğan & Erdoğan, 2013).

Self-reports were administered both individually (*n* = 23) and in a group (*n* = 37), but in 23 of the identified self-reports, this distinction was not specified in the studies’ methodological descriptions. Surveys and questionnaires were mainly administered in whole-class groups (*n* = 31), whereas interviews were often administered individually (*n* = 20) or in focus-groups (*n* = 2). Although most of the student interviews were conducted by the researchers at school, some were also conducted in the students’ homes (e.g., Abbott, 2000).

#### Adapting Previously Used Self-Reports

Out of the 83 identified self-reports, authors chose to employ previously used motivational measures 41 times, either without modifying them (*n* = 10), or by adapting them to the needs of the studies (*n* = 31), whereas authors developed new measures for their investigations 42 times. In less than 15% of the times when interviews were used (*n* = 7), authors indicated that interview guides were developed based on previously used questions. In contrast, authors often used surveys and questionnaires that had previously been used (*n* = 34), either without modifying them (*n* = 10), or with modifications (*n* = 24). With regards to alternative written responses, authors did not indicate whether they were adaptations of previously used motivational measures.

Self-report modifications included linguistic adaptations, such as translations (e.g., Babayigit, 2019; Nicolaidou, 2012) and changes in the wording of items to account for age-adequate content (e.g., Hier & Mahony, 2018). In addition, items were also modified from different domains to be applicable to writing, for instance, by adapting items from reading to writing (e.g., Kanala et al., 2013). However, in some cases, it is not always clear what these modifications entail, for example, when authors state that part of the items from a specific scale is used, but do not specify which items (e.g., Göçen, 2019).

#### Items and Responses

A variety of items were used in the studies to measure motivation constructs. Items included both statements and questions, and students could provide their responses by answering open-ended questions or through marking options in close-ended questions.

Open-ended questions were most commonly used in student interviews and often asked students about their self-beliefs (e.g., “What do you do really well as a writer?” from Hillyer & Ley, 1996) and preferences (e.g., “Are there any things in particular you like to write about?” from Nolen, 2007). In surveys, open-ended questions were often used to ask students to provide reasons to their answers in close-ended questions (e.g., Silver & Lee, 2007).

Close-ended scales varied from two-tiered frequency responses divided between “always” and “usually” (Wilson & Trainin, 2007) to 10-point Likert scales ranging from “1 = not very sure” to “10 = very sure” (Miller & Meece, 1999). Some surveys consistently used the same close-ended responses for all of the items in the survey (e.g., Bayat, 2016). However, some surveys combined different response scales, depending on the items. For instance, in Akyol and Aktas (2018), although most of the items are judged by the students through a 4-point Likert scale, the responses include both frequency scales from “almost never” to “always,” and evaluation scales from “a poor author” to “a very good author,” as well as its inverted sequencing from “very good” to “poor.”

Given the young age of the students being investigated, in 19 of the studies researchers also chose to use stuffed animals or images to support the students in their responses. Stuffed animals were used three times for different purposes. In Mata (2011), two different stuffed animals represented contradictory statements, and kindergarten children could choose if they were “a little” or “a lot like” the chosen stuffed animal for each item, which resulted in a 4-point Likert scale. In Nolen (2007), a monkey hand puppet was used to ask children in first grade to describe reading and writing in school, and in Schrodt et al. (2019), it is not specified how the stuffed animals were used in the study. When it comes to images, although Garfield, the cat was used most often (*n* = 6) (e.g., Paquette, 2008), a variety of other symbolic images was also used in all grades to represent different responses in Likert scales. These included, for instance, dogs (e.g., Paquette et al., 2013), teddy bear faces (Merisuo-Storm, 2006), boxes (e.g., Hier & Mahony, 2018), and happy and sad faces (Jones et al., 2016). In Table 5, we present some examples of the items used in the studies to measure the five theorized components of motivation, and samples of both open- and close-ended responses.

**Table 5:** Examples of motivation constructs addressed in items and responses

Theorized component		Open-ended			Close-ended	
		Sample item	Sample response		Sample item	Sample response
Motivation	Abbott (2000)	Why do you choose to write?	“I don’t know. I just like to write. I know if you write enough, then you’ll get better and better. […]”	Li and Chu (2018)	“I want to spend more time in writing because of using “Joyous Writing Club.”	5-point Likert scale with 1 = strongly disagree and 5 = strongly agree
Self-beliefs	Nicolaidou (2012)	Do you think your essays in your portfolio show progress?	Yes, I believe that they do	Grenner et al. (2020)	- I can quickly write a text on the computer - I can read through my text and correct spelling mistakes - I can read through my text and make changes to improve it	Beneath each statement was a 100-mm, visual-analogue scale. The VAS scale was marked with the phrases “not at all” and “yes, completely” below the left and right endpoints. One of the researchers explained how students should mark the scales according to their beliefs, with illustrations on the whiteboard
Goal-orientations	Hillyer and Ley (1996)	What would you like to do better as a writer?	“I need to improve on using quotation marks correctly in my stories. I’m not sure how to use them when I let my characters have conversation.”	Miller and Meece (1997)	*Task-mastery* item example: “I wanted to learn as much as possible on this assignment.” *Ego-social* item example: “I wanted the teacher to think I am doing a good job on this assignment.”	Students rated on a 4-point scale according to how well the statement described how they completed an assignment (1 = not at all to 4 = very much)
Interest and value	Chen and Liu (2019)	Think and describe your attitude towards. Does your writing attitude change before and after the use of the 4S approach? Why or why not?	“Before using the 4S approach, I initially thought that writing and composition were both difficult for me. Those two tasks are interesting and now I am more willing than before to share stories with my classmates. In addition, the story structures exhibited in the tasks are clear and they are useful when I am writing the story.”	Babayigit (2019)–Adapted from Graham, Berninger and Fan (2007)	“How do you feel” … “ about writing for fun at home”, “when you write in school during free time”, “about writing during summer vacation”, “about writing instead of playing”, “about writing in school”, “about spending free time writing”, and “when it’s time for writing at school.”	Students indicated their attitude by marking one of four images of Garfield the Cat, ranging from very happy (score of 4) to very unhappy (score of 1)
Outcome attributions	Truax (2018)	“When I work hard at my writing, my writing gets better” Strongly disagree = 1, Disagree = 2, Agree = 3, Strongly agree = 4 Explain why you think that	“If I do hard work, it gets better, and I’ll make good books.”	Hier and Mahony (2018)	“Did you work as hard as possible?” “Did you try to do a good job writing your story?”	Students responded to items on a five-point, Likert-type scale. Response options were presented as boxes with the words not at all, a little bit, some, a lot, and very, very much under each one. The box above each response option became successively larger from not at all through very, very much to denote the relative level of agreement each option represented

#### Why is Validation of Self-Reports of Motivation to Write Important?

The results above show that new unvalidated self-report measures are used extensively in the research targeting writing motivation in early elementary education. Interestingly, out of the 83 identified self-report measures identified in the present review, previously used motivational measures were used 41 times, either with or without modifications. This means that 42 times, authors developed new measures for their studies. This preponderance of newly developed instruments documented in the present review is symptomatic of the claimed limbo state of writing motivation research, and therefore worthy of attention and reflections. According to Haladyna et al. (2002), new measures represent the most common validity threats in the field of assessment. Although validity in testing refers to the accurate and meaningful interpretation of test scores and the reasonableness of the inferences drawn from test scores (AERA et al., 2014; Messick, 1998), most threats for valid interpretations relate directly to the quality of the constructed measures (Cook et al., 1979). That is, with a preponderance of poorly crafted tests, misinterpretations and inappropriate uses of test score data are likely. The use of unvalidated measures also seemed evident in studies investigating motivation to write using student interviews and alternative written responses, as they often did not present a rationale for the construction of these measures.

However, although we see the value of creativity and innovation, new measures should be accompanied by pilot testing and discussions of validity, and in failing to do so, instead they threaten to hamper scientific progress. In total, we recognize that creativity is truly needed in assessments of writing motivation but should be combined with sound measurement development to form a solid basis for this evolving field of research. To do this, it should be mandatory that researchers define the motivation constructs explored in their studies, as remarked by Camacho et al. (2021), and make explicit their rationale for choice/design of measure of motivation. Only then, can measures be scrutinized by the research community and end users, for scientific purposes or classroom uses.

#### Does It Matter What Kind of Pictorial Support We Use in Self-Report Measures?

The format of scales designed for children is an important area to consider, to confirm we are on the right track when measuring something. Specifically, we must acknowledge the role of pictorial support—e.g., typical scales including faces with different moods—as this format is most frequently applied to elicit student thinking in the studies included in the present review. Pictorial support is a recommended practice when measuring young children’s motivation (Bandura, 2006), as an aid to capture their perspectives, especially if they lack the necessary vocabulary to express themselves. This broad guideline is a relatively uncontroversial recommendation in the field, but exactly what type of pictorial support to use is far less unanimous.

More precisely, a further finding in the current study is that a myriad of pictorial supports was used, including stuffed animals and picture-scales of different cats and dogs, and in most cases objectively constructed out of convenience. This diversity is somewhat surprising in the field of motivation, as proponents of this field are likely to have a particular sensitivity to how nuances in external factors may trigger humans’ inner drives differently. Returning to the focus on validity, the spectrum of different varieties of pictorial support revealed in this study can be seen as uncontrolled variables which may erroneously inflate or deflate scores for some or all examinees. Such uncontrolled variables can lead to construct-irrelevant variance—reducing the accuracy of test score interpretations and thereby the validity of the test in question (Cook et al., 1979; Messick, 1998).

The array of unvalidated pictorial supports is particularly problematic, given the early advice of Bandura (2006, p. 313) to use *circles* with progressively larger sizes for representing students’ increasing confidence that they can perform the tasks addressed in the measures. In this, Bandura clearly stated that *happy or sad faces* in self-efficacy scales should be avoided, because children may misread such scales as measuring their emotional states rather than how confident they are that they can perform given tasks. Bandura’s caution about the use of pictorial scales is based on a clear rationale, and ought to be validated as a starting point for investigating the convenience of other ways to provide pictorial support for young students. None of the studies included in the present review refers to the guidelines of Bandura (2006) or other relevant guidelines for their use of pictorial support in scale construction.

### What Emphasis Is Given to Students’ Voices in the Studies?

#### Students’ Self-Reports Versus Reports from Others

Although every study in this review included at least one measure of motivation based on *students’ self-reports*, the degree of emphasis placed on the students’ voices when reporting results and findings varied considerably among the studies. Mainly, this difference was a consequence of the type of data used in the studies. That is, students’ voices were given a predominant role in some studies as they only included students’ self-reports as their primary data (e.g., Mata, 2011; Seban & Tavsanli, 2015). However, other studies combined the students’ self-reports with reports from teachers (e.g., Li & Chu, 2018), researchers (e.g., Hall et al., 2017), and parents (e.g., Teague et al., 2010), which would juxtapose the students’ voices with the viewpoints of others.

In studies where data from students’ self-reports were triangulated with teachers’ and/or researchers’ observations/evaluations, it was sometimes unclear what contribution each source had for the findings, as they were not clearly presented in the results. For instance, although Hertz and Heydenberk (1997, p. 205) state that “informal and formal assessments, observations of students’ writing process behaviors, and parent, teacher, and student interviews” were used for data collection, these are not presented separately in the results. When findings are discussed, it is therefore difficult to pinpoint how much the students’ own viewpoints contributed to the studies’ findings.

In addition, whereas some studies provide examples of items and students’ responses (e.g., Erdoğan & Erdoğan, 2013; Snyders, 2014), others provide only examples of the questions asked to students, without explicitly providing examples of what the students actually answered, which again makes it difficult to gain a better insight of the students’ perspectives. For instance, in Lee and Enciso (2017), the open questions “What is good writing?” and “What does a good writer do?” were included in their survey, but examples of student answers were not provided.

In other cases, even if results are presented separately, the students’ responses may sometimes play a smaller role in the findings than the teachers’ evaluations. For example, after analyzing data from both teachers’ and students’ responses, Chohan (2011, p. 39) recommends the implementation of a schoolwide mailing program, arguing that “data analysis indicated that children enjoyed the responsive letter writing process.” However, although qualitative results from teachers’ evaluations suggested that this intervention had contributed positively to students’ writing motivation, quantitative data from student surveys indicated otherwise.

#### Measuring More Than One Domain

Determining the students’ perspectives is also important in studies measuring other domains, in addition to writing. For instance, in seven of the included studies, reading motivation was also investigated, and in one of the studies (Hall et al., 2017), it was difficult to ascertain how interested students were in writing, as most of the information on interest derived from students’ self-reports were about reading, while results regarding motivation to write seemed to be mostly derived from observations and reports from teachers and parents. In addition, looking at the results derived from all 56 studies, students seem to have high levels of motivation to write. However, in five of the seven studies that measured both reading and writing motivation, students demonstrated higher levels of motivation towards reading, rather than writing, which could indicate issues related to conformity in the self-reports. That is, when only asked questions about writing, students might provide positive answers as they think this is what is expected of them, whereas when asked about different domains, they might feel “freer” to be honest about each domain separately. At the same time, when asked about their motivations towards different domains, students might also feel that choosing their favorite is necessary, which might influence their choices. Whether measuring only one domain or more than one thus needs deliberate consideration.

#### Combining Multiple Sources of Student Self-Reports

Even in studies that only included students’ self-reports, these were sometimes derived from different data sources, such as multiple surveys (e.g., Göçen, 2019; Seban, 2012) or surveys and interviews (e.g., Kholisiyah et al., 2018; Nicolaidou, 2012; Perry, 1998), and the role that each data source played in the studies also varied. For instance, Truax (2018) combined quantitative data from students’ surveys with qualitative data from students’ interviews, and although quantitative results indicated that teacher’s growth mindset feedback had no statistically significant effects, qualitative findings suggested that objective feedback positively impacted writing motivation.

Combining quantitative and qualitative methods was commonly used for complementary purposes, where quantitative methods, such as scales, were used to measure *frequencies* and *levels*, that is, *how often* students worked with writing activities and *how much* they enjoyed or valued these activities. Qualitative methods alone, such as open-ended questions in interviews and surveys, were used to investigate *reasons* for their choices and *factors* that influenced those choices. However, although some studies only investigated frequencies and levels, such as how much students were motivated to write, often in the studies’ discussion section, authors also argued about reasons and factors for the students’ answers. For instance, after analyzing quantitative data from three different scales, Göçen (2019) concludes that creative writing activities had a positive effect on students’ creative writing achievement, writing attitude, and motivation. The author then suggests in the implications section of the article that “it is necessary to adopt a process-based writing approach and conduct creative writing activities in teaching mother tongue in order to enable students to advance their writing skills, develop positive attitudes towards writing and enjoy writing” (Göçen, 2019, p. 1038). Nevertheless, the scales used in the study only measured *whether* motivation levels increased, rather than *why* they would increase. In such cases, questions about reasons and factors should be explicitly asked.

Finally, studies also combined different sample sizes, depending on the type of data collected. Commonly, larger groups responded to surveys, and smaller (mostly purposeful) samples participated in interviews (e.g., Bayraktar, 2013). For example, in Nair et al. (2013), a larger sample of 197 students responded to a survey, whereas a smaller purposeful sample of 12 students who did not submit their online assignments, and 6 who did it, participated in interviews to give reasons that affected their motivation to finish and submit (or not) their assignments.

#### Presenting Results from Each Data Source and Reporting Findings

In sum, the present analysis shows in what ways students’ self-reports were weighted differentially across studies. That is, students’ voices were given more prominence when students’ self-reports represented the primary data source (e.g., Mata, 2011). However, students’ perspectives were often given less prominence when combined with other data sources, such as reports from teachers (e.g., Li & Chu, 2018), researchers (e.g., Hall et al., 2017), and parents (e.g., Teague et al., 2010). Therefore, future studies should be aware when presenting results on students’ motivation from different data sources, i.e., publications should clearly state *who* has uttered *what* and further on, from *whose voices* the conclusions are drawn. Frameworks for guiding the integration of mixed methods data provide guidance in this area (Fetters et al., 2013). For clarity purposes, although analyses combine and synthesize different data sources, data for each source should be presented separately, and examples of items/questions *and* responses should be provided alongside the publication.

### Limitations

The present review has four main limitations. First, even though comprehensive database and hand searches were conducted to identify relevant studies, we acknowledge that not all available studies were captured in this review as a result of stringent inclusion criteria. For instance, only including studies published in English give more prominence to research derived from specific countries and may overlook relevant advancements in the assessment of writing motivation originating from other parts of the world. In addition, to lessen limitations related to the quality of included studies, like Camacho et al. (2021) and Miller et al. (2018), the present review only includes peer-reviewed studies, as these studies have undergone the rigorous demands of the peer-review process, which is generally accepted “as a method to ensure a level of academic credibility” (Miller et al., 2015, p. 467). However, even though relevant as a quality threshold, this criterion can be a source of bias. For instance, as documented by Polanin et al. (2016), the presence of publication bias in education and psychology can lead to skewed effect sizes in meta-analyses. Nevertheless, publication bias does not represent the same type of threat in the present review, given that the way the RQs of our review are formulated—partly resembling the ones of a scoping review instead of a meta-analysis—points rather to sampling in order to achieve saturation. As such, by purposively sampling studies from peer-reviewed journals that purportedly reflect research trends and standards in writing motivation, we argue that the studies included in our review provide the variability necessary for investigating the RQs addressed in our study.

Second, we acknowledge that ending the search in April 2020 excludes studies published after the occurrence of the COVID-19 pandemics, which may have influenced students’ motivation and perhaps how writing motivation was measured during lockdown periods. We acknowledge this limitation and encourage researchers to explore whether such a change has occurred, which would be an interesting starting point for future reviews; however, this question is beyond the scope of the present study.

Third, inconsistencies of construct definitions in writing motivation coupled with the use of a diverse range of self-report practices limited our ability to synthesize findings towards specific standards for designing writing motivation self-reports. However, we provide recommendations that can serve as initial suggestions towards more standardized practices.

Finally, we focus specifically on self-reports used with K-5 students, but it is possible that a systematic review investigating older students could reveal more uniform practices and guidelines, which in turn could potentially inform the design of self-reports for younger students. However, to the best of our knowledge, no such reviews have been conducted, hence we could not compare our findings with those for older students.

## Conclusion and Future Directions

Particularly in the study of writing motivation, researchers must be sensitized to the target of the motivation—the writing task itself. The greater construct of “writing” represents an array of genres and formats, and each type is endowed with potential challenges and joys. Therefore, how writing is presented can greatly impact how a young writer responds. We must not assume that because a young writer is unmotivated to compose a creative story that they are unmotivated towards the larger construct of “writing.” This is akin to assuming that, for example, if a student did not want to play a game of dodgeball they did not like athletics. In the reviewed studies, an over-simplification or narrow operationalization of writing represented a significant validity risk to the study of writing motivation. However, this also provides an important opportunity to course-correct and strengthen the rigor of research in this area.

Findings from the present study show extensive—some would even say reckless—use of unvalidated self-report measures of motivation to write in the early grades. New measures should be solidly anchored in theory of motivation and writing, and systematically piloted before implementation in classrooms or for research purposes. In particular, pictorial support in measures of writing motivation should follow existing guidelines for scale construction (e.g., Bandura, 2006).

Furthermore, in our quest for better understanding students’ motivation to write, listening to their own perspectives on this matter is an important step towards building this understanding. However, the role that this rich source of data plays when analyzing findings needs to be clearly delineated. That is, especially in studies combining the students’ views with the perspectives of others, such as teachers and parents, researchers need to report clearly whose perspectives are being portrayed and what role they play in the studies’ findings. In addition, to strengthen transparency of how the students’ reports are evaluated, researchers should provide examples, not only of the *items* asked, but also of *responses* uttered by the students.

The conditions highlighted above all pose threats to validity in different ways, as validity is defined as “The degree to which evidence and theory support the interpretation of test scores for proposed uses of tests” (AERA et al., 2014, p. 1). The emphasis on interpretation and use underscores that validity exceeds the boundaries of the test itself (i.e., psychometric qualities), rendering a situation where validity in the groundbreaking sense is dependent on intended and appropriate use of test scores. The lack of a consistent framework of constructs of motivation to write, as shown in this study, will continue to pose validity threats until solved.

Over a long period of time, assessment researchers have emphasized challenges of reliability and psychometrics over challenges posed by construct-underrepresentation. The lack of attention towards the different writing genres used when investigating motivation to write therefore represents a clear construct-underrepresentation in current research base on motivation to write. Argument-based validity (Kane, 1992, 2006) emphasizes the importance of starting validation from aspects of interpretation and use. An obvious starting point would be to listen to the students’ own voices: “If we want to know what is actually in the interest of the child, it is logical to listen to him or her” (UNCRC, 1989; article 12:1). According to established definitions, this is also the most likely place from which constructs should be “constructed,” as constructs are theoretical and not directly observable (Thorndike & Thorndike-Christ, 2014, p. 135). This is also why Chapelle (2020) claims that argument-based validation is an intended means for bridging the gap between theory and practice.

Our findings may provide some initial suggestions for approaching the limbo state of the field of motivation to write, as addressed above. Future research and development should (1) work to counteract existing biases in writing tasks, (2) provide a rationale for their choice/design of measure of motivation, and explicitly state what the chosen instrument is measuring, and (3) report clearly whose voices are being heard (e.g., students’, teachers’, or researchers’) and the appropriateness of this choice regarding study purpose, design, and findings.

## Supplementary Information

Below is the link to the electronic supplementary material.

Supplementary file1 (PDF 39 KB)

Supplementary file2 (PDF 40 KB)
