# Supplementary material for: Assessing Writing Motivation: a Systematic Review of K-5 Students' Self-Reports
Source: Educ Psychol Rev. 2023 Feb 23;35(1):24. doi: 10.1007/s10648-023-09732-6 (PMC9947433; doi:10.1007/s10648-023-09732-6)
Supplement: Supplementary file 1 — Supplementary file1 (PDF 39 KB) [file 10648_2023_9732_MOESM1_ESM.pdf]

Figure S1
Flow Diagram of Article Selection Process.

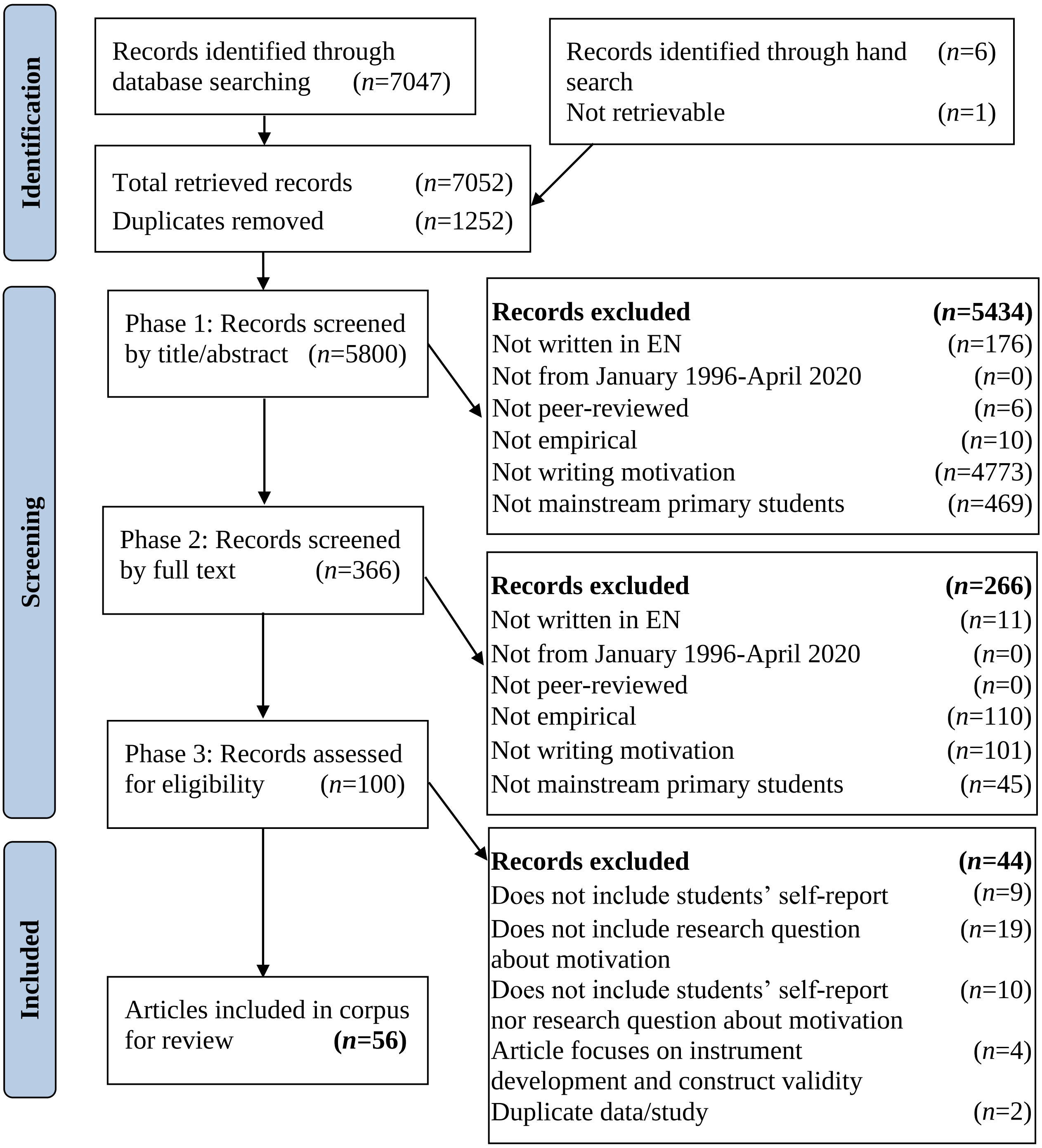

Adapted from: Page, M. J., McKenzie, J. E., Bossuyt, P. M, Boutron, I., Hoffmann, T. C., Mulrow, C. D., et al. (2021) The PRISMA 2020 statement: an updated guideline for reporting systematic reviews. BMJ 2021;372:n71. doi: 10.1136/bmj.n71
