# Supplementary material for: Assessing Writing Motivation: a Systematic Review of K-5 Students' Self-Reports
Source: Educ Psychol Rev. 2023 Feb 23;35(1):24. doi: 10.1007/s10648-023-09732-6 (PMC9947433; doi:10.1007/s10648-023-09732-6)
Supplement: Supplementary file 2 — Supplementary file2 (PDF 40 KB) [file 10648_2023_9732_MOESM2_ESM.pdf]

Table S1
Overview of Included Studies

Study	*Country*	*Grade level(s)*	*Student sample size*	*Methodology*	*Type of student self-report(s)*	*Motivation construct*	*Type of writing task*	*Main findings*
Abbott (2000)	USA	5th	2	Qualitative	Interviews	Intrinsic motivation	N/A	Interpretive study of two avid writers showed that they describe flow experiences similarly to older students and that the classroom social context influenced their intrinsic motivation to write.
Akyol & Aktas (2018)	Turkey	4th	185	Quantitative	Survey	Task value<br>Self-efficacy<br>(The survey uses the term *self-concept*, but the discussion uses *self-efficacy*)	Task: Story-writing<br>Survey questions about narrative, expository and general writing	As the value placed on writing by students and their perception of themselves as competent writers increase, their story-writing scores also increase.
Andrzejczak et al. (2005)	USA	2nd and 3rd	2	Qualitative	Interviews	Motivation	Poetry, narrative, or expository writing	Visual art creation enhances the writing process and artwork as a pre-writing activity becomes a motivational entry point.
Babayigit (2019)	Turkey	4th	35	Quantitative	Survey	Attitude	Creative writing	Although creative writing activities led to an increase in reading, writing and language lesson attitude, only increase in language lesson attitude was significant.
Bayat (2016)	Turkey	4th	42	Quantitative	Survey	Attitude	Creative writing (letters, stories, tales, fables, poems)	Students receiving a creative writing program based on speaking activities increased their writing attitudes more than students in the control group.

Study	*Country*	*Grade level(s)*	*Student sample size*	*Methodology*	*Type of student self-report(s)*	*Motivation construct*	*Type of writing task*	*Main findings*
Bayraktar (2013)	USA	5th	22	Mixed methods	Survey with entire sample for purposive sampling of 4 students to be interviewed	Self-efficacy<br>Enjoyment	Student-teacher writing conferences about diverse genres (e.g., historical fiction, expository text, and persuasive essay)	Students' interactions differed during student-teacher writing conferences, depending on their levels of writing self-efficacy. Students with lower levels displayed weaker commitment to their tasks, and had difficulties in sharing their writings and seeking their teacher's help. Students with higher levels were more self-disciplined and committed to their writing goals, and were not hesitant in discussing their writings and seeking help when needed.
Beck & Fetherston (2003)	Australia	3rd	7	Qualitative	Interviews	Attitude	Story writing on paper and in a word processor<br>(Students were given story starters)	Student self-reports indicated that the incorporation of a word processor influenced positively the students’ enjoyment of the writing process as it facilitated the completion of writing tasks and the editing process.
Boscolo et al. (2012)	Italy	4th	114	Mixed methods	Survey and questionnaire with open-ended questions	Liking<br>Self-perception of competence	Linguistic games and narrative writing	Students in an intervention teaching them to play with narrative texts reported that they liked writing more at posttest compared to students in a business-as-usual condition, however no effect on their perceived competence was found.

Study	*Country*	*Grade level(s)*	*Student sample size*	*Methodology*	*Type of student self-report(s)*	*Motivation construct*	*Type of writing task*	*Main findings*
Boyaci & Güner (2018)	Turkey	4th	46	Mixed methods	Survey and semi-structured interviews	Motivation	Authentic writing	The use of authentic task-based material improved the reading comprehension, writing skills and writing motivation of the students.
Bradford et al. (2016)	USA	1st and 2nd	32	Mixed methods	Survey including one open written response and a final reflection essay on the use of rubrics	Attitude	Opinion paragraphs in response to given writing prompts	Although an intervention focusing on the use of rubrics did not show a significant difference between conditions according to student attitude ratings, student-reported reasons indicated that the use of rubrics promoted positive attitudes toward writing later and higher self-efficacy.
Chen & Liu (2019)	China	5th	49	Mixed methods	Two questionnaires and semi-structured interviews	Attitude<br>Interest	Story writing	Findings indicated that the 4S approach increased students' story quality, attitudes, and interest.
Chohan (2011)	Canada	1, 2, 3, 4, and 5	122	Mixed methods	Survey including open-ended questions	Attitude<br>Perceptions of themselves as writers	Schoolwide letter writing	Although the number of students who indicated that they should write letters at school to each other almost doubled, the intervention did not show a significant impact on their attitudes toward writing.
Erdogan & Erdogan (2013)	Turkey	5th	594	Qualitative	Students' completion of the metaphor "writing is like…"	Perception about writing	Completion of metaphorical sentence: ''Writing is like… because…''	Students' metaphors were sorted under 14 categories, like writing is 'joyful', 'informative', and 'developing', and results suggested that students mostly conceptualize writing as an activity that they like, rather than something boring.

Study	*Country*	*Grade level(s)*	*Student sample size*	*Methodology*	*Type of student self-report(s)*	*Motivation construct*	*Type of writing task*	*Main findings*
Gallini & Zhang (1997)	USA	4th and 5th	84	Mixed methods	Survey and discussion sessions	Confidence<br>Self-efficacy	Writing about real-world science	Students who preferred group work enjoyed interaction and receiving input from peers. Higher audience awareness indicated greater orientation towards group work and the use metacognitive skills in writing, whereas students who preferred individual work showed higher self-efficacy rates and marginal significance in using metacognitive skills. Lower ratings of confidence in writing were more present among those with less audience awareness.
Graham et al. (2012)	USA	1st and 3rd	241	Quantitative	Survey	Attitude	Task: Personal narrative<br>Survey questions about writing at school and at home	Students' ratings indicated that reading and writing are separable constructs, but some aspects like literacy interactions might not be. No statistically significant correlations between SES measures and attitude measures were found.
Grenner et al. (2020)	Sweden	5th	55	Quantitative	Survey	Self-efficacy	Personal narrative	Students' pre-test ratings indicated strong self-efficacy for narrative writing, which increased after intervention. Moderate correlations between self-efficacy and writing performance were found pre- and post-intervention.

Study	*Country*	*Grade level(s)*	*Student sample size*	*Methodology*	*Type of student self-report(s)*	*Motivation construct*	*Type of writing task*	*Main findings*
Göçen (2019)	Turkey	1, 2, 3, and 4	630	Quantitative	Three different surveys	Attitude<br>Motivation	Creative writing	Findings indicated a positive effect of creative writing on students' creative writing achievement, writing attitude and motivation.
Hall & Axelrod (2014)	USA	K, 1, 2, 3, 4, and 5	81	Qualitative	Focus group interviews	Attitude<br>Self-efficacy (including students' feelings and emotions for writing)	N/A	Students who perceived their teachers as enthusiastic in their practices and towards writing expressed positive writing attitudes. Students also expressed preferences regarding writing, including choice of topic, genre, writing for different purposes and audiences, and whether sharing their writings or not.
Hall et al. (2017)	USA	Kindergarten	41	Mixed methods	Interviews	Interest	Informational text	After using The Tools Approach strategy, children increased their abilities to identify and use informational text features. Their interest in reading and writing this type of text also increased after the intervention.
Hertz & Heydenberk (1997)	USA	Kindergarten	19	Mixed methods	Interviews	Attitude	Story writing	Findings indicate that process writing instruction increased students' writing skills, and that student motivation was positively influenced by the interactive format of the intervention.

Study	*Country*	*Grade level(s)*	*Student sample size*	*Methodology*	*Type of student self-report(s)*	*Motivation construct*	*Type of writing task*	*Main findings*
Hier & Mahony (2018)	USA	2nd	117	Quantitative	Survey	Effort<br>Self-efficacy	Narrative and expository writing	After participating in a performance feedback writing intervention, students reported higher levels of writing self-efficacy. Although effort was a significant predictor of writing self-efficacy, their experiences with task mastery and positive feedback were not.
Hillyer & Ley (1996)	USA	2nd	32	Qualitative	Interviews	Perceptions of themselves as writers	Different types of writing to be included in portfolios and self-assessments	A portfolio-based intervention showed positive effects on students' perceptions of themselves as writers; their ability to set goals for their literacy development, and to self-assess their writings and their progression toward their goals.
Ihmeideh (2015)	Jordan	Kindergarten	46	Mixed methods	Interviews	Attitude	Different types of functional writing (e.g., shopping lists, prescriptions, cards, and messages)	After the integration of writing activities into a dramatic play center with child-size furniture and real-life scenarios, such as grocery shops and doctor offices, children demonstrated significant improvement in their writing skills and attitudes.
Jones et al. (2016)	USA	1st and 2nd	42	Quantitative	Questionnaire	Interest	Spelling practice through rainbow writing and quizzing (retrieval practice)	Both retrieval practice and rainbow writing promoted student learning, but retrieval practice led to better results. Students indicated enjoying retrieval practice as much or more than rainbow writing.

Study	*Country*	*Grade level(s)*	*Student sample size*	*Methodology*	*Type of student self-report(s)*	*Motivation construct*	*Type of writing task*	*Main findings*
Kanala et al. (2013)	Finland	5th	25	Mixed methods	Questionnaire with multiple choice and open-ended questions	Motivation	Writing in a mobile app	Results indicated that the use of a mobile application to write showed a moderate positive effect on the students' motivation to write.
Kholisiyah et al. (2018)	Indonesia	5th	18	Mixed methods	Survey and interview	Achievement motivation	Explanatory text	Results indicated that outstanding students display high levels of writing achievement motivation and that their motivation is influenced by individual (e.g., competence and beliefs) and environmental factors (e.g., competitive environments, parent support and teachers).
Kim & Lorsbach (2005)	USA	K to 1st	18	Qualitative	Interview	Self-efficacy	Story writing by completing a sentence like "To be a good friend, I can…"	Despite their young age, children were able to indicate their levels of writing self-efficacy. Their ratings mostly showed consistency with teachers' and researchers' ratings, and differences seemed to be related to the students' definitions of writing.
Lee & Enciso (2017)	USA	3rd	29 classrooms	Quantitative	Survey	Self-efficacy	Story writing following a story spine	An intervention with teaching artists from a theater increased students' self-efficacy for pre-writing and also the students' skill and confidence in idea generation and revision.

Study	*Country*	*Grade level(s)*	*Student sample size*	*Methodology*	*Type of student self-report(s)*	*Motivation construct*	*Type of writing task*	*Main findings*
Leroy (2000)	Canada	5th	1	Qualitative	Interview	Attitude	Creative and journal writing	Although the girl in the study expressed enjoyment in writing poems and stories in class, and believed that the purpose of these was to entertain her readers, she complained that writing responses to her teacher about the books and texts she had read ruined the joy of the reading activity.
Li & Chu (2018)	China	4th	109	Mixed methods	Questionnaire, individual interviews, and focus group interviews	Motivation	Collaborative writing in a wiki	Students were mostly positive about the collaborative process writing through wikis, but reported on learning difficulties related collaborations and the wiki-platform.
Liao et al. (2018)	Taiwan	3rd	245	Quantitative	Survey	Individual and situational interest	Writing in a game-based environment vs. online-based environment	Students writing in a game-based environment showed greater situational interest and were more motivated to generate ideas and write longer articles than students writing in an online-based environment.
Mata (2011)	Portugal	Kindergarten	451	Quantitative	Survey	Enjoyment<br>Value<br>Self-concept	Survey questions about writing stories	Although students indicated high levels of motivation for both reading and writing, motivation levels for reading were higher. Reading and writing value scores were higher than enjoyment and self-concept.
Merisuo-Storm (2006)	Finland	4th	145	Quantitative	Survey	Attitude	N/A	Students reported more negative attitudes towards writing than towards reading.

Study	*Country*	*Grade level(s)*	*Student sample size*	*Methodology*	*Type of student self-report(s)*	*Motivation construct*	*Type of writing task*	*Main findings*
Miller & Meece (1997)	USA	3rd	187	Quantitative	Survey	Goal orientations<br>Cognitive engagement<br>Anxiety<br>Self-concept	Complex writing tasks	In classrooms where students had more opportunities to work with challenging writing tasks, write collaboratively, and self-monitor their progress over time, they demonstrated more positive motivational patterns.
Miller & Meece (1999)	USA	3rd	24	Mixed methods	Interview where students answered a survey by rating their answers from 1 to 10, and were also asked open-ended questions to explain their answers	Performance expectancies<br>Task value	High-challenge tasks (e.g., essays on a topic of students' choice, research papers on an animal for an interdisciplinary science unit, a character analysis for a class novel, letters to politician describing election concerns, and letters to the next year's third-grade class explaining what students needed to know in their new grade) and low-challenge tasks (e.g., worksheets on unstressed vowels, pronouns, simple contractions, and vocabulary, spelling, and handwriting exercises)	In general, students preferred high-challenging writing tasks, rather than low-challenging, as these were seen as boring and not intellectually challenging. Those who did not have enough opportunities to work with challenging tasks, questioned whether they had the necessary skills.
Nair et al. (2013)	Singapore	5th	224	Mixed methods	Online survey with entire sample and interviews with 18 students	Motivation	Expository text and blogging	Although most of the students agreed that blogging was useful, important and interesting, both students and teachers tended to take traditional paper-based writing assignments more seriously than online-based.

Study	*Country*	*Grade level(s)*	*Student sample size*	*Methodology*	*Type of student self-report(s)*	*Motivation construct*	*Type of writing task*	*Main findings*
Nicolaidou (2012)	Cyprus	4th	146	Mixed methods	Two different surveys with entire sample and semi-structured interviews with 9 students	Self-efficacy	Process portfolios	Results indicated that portfolio affordances connected to sources of self-efficacy, like verbal persuasion, and mastery and vicarious experiences, influenced positively the students' writing self-efficacy.
Nolen (2007)	USA	1, 2, and 3	67	Mixed methods	Interviews	Interest<br>Motivation	N/A	Results indicated that the classroom social context played an essential role for the children's motivation to read and write.
Paquette (2008)	USA	2nd and 4th	85	Mixed methods	Survey and interviews	Attitude	Various writing tasks	Survey results did not indicate significant differences between treatment and control groups. However, even though second-graders lacked language skills to express themselves accurately, interview answers from second and fourth graders indicated that students appreciated the cross-age tutoring program, despite collaboration issues.
Paquette et al. (2013)	USA	K, 1, 3, 4, and 5	133	Mixed methods	Survey including 3 open-ended questions	Attitude	Various writing tasks during Drop Everything and Write sessions, including personal written communication (note passing), free writing (creative, personal narrative, expository writing), and prompt (personal response to an event or literature).	Although teachers' observations of students' behaviors indicated that the implementation of Drop Everything and Write was successful, students' survey data were contradictory.

Study	*Country*	*Grade level(s)*	*Student sample size*	*Methodology*	*Type of student self-report(s)*	*Motivation construct*	*Type of writing task*	*Main findings*
Perry (1998)	Canada	2nd and 3rd	94	Mixed methods	Questionnaire including open-ended questions and interviews	Control<br>Support<br>Beliefs<br>Values<br>Expectations	N/A	Students in classroom contexts characterized by high self-regulated learning demonstrated more positive motivational orientations.
Perry et al. (2003)	Canada	1st	2	Mixed methods	Interviews	Motivation	Task: Story writing<br>Interview questions about story writing and spelling.	Although both students indicated constructive views on failure, the high achieving student was more negative towards errors. Both had strategies for coping with challenging writing tasks, but the lower achieving preferred less challenging tasks.
Pollington et al. (2001)	USA	4th and 5th	130	Quantitative	Survey	Self-perception	Writing during workshop vs. traditional writing instruction	There were no significant differences on the students' scores on writer self-perception between students receiving writing workshop instruction and those receiving traditional instruction. Findings suggest that teachers are more decisive in affecting the students' writer self-perception.
Schrodt et al. (2019)	USA	Kindergarten	27	Mixed methods	Interviews, survey, and recorded conversations during measurement tasks	Motivation (including questions about, attribution, competence and difficulty, self-efficacy, value and enjoyment, and mindset)	Enhanced writer's workshop	Quantitative and qualitative results indicated that adding instruction on mindset and self-regulation to the writer's workshop improved the students' writing motivation and independence.

Study	*Country*	*Grade level(s)*	*Student sample size*	*Methodology*	*Type of student self-report(s)*	*Motivation construct*	*Type of writing task*	*Main findings*
Seban (2012)	Turkey	3rd	42	Quantitative	Two different surveys	Attitude<br>Self-perception	Picture and poem books	The authoring cycle program had a positive effect on the experimental group's attitudes toward writing, but no significant effect on their self-perception as writers, compared to the control group. No significant differences were found between the groups' writing achievement, as measured in terms of appearance, organization, development, language, and length of composition.
Seban & Tavsanli (2015)	Turkey	2nd	27	Qualitative	Interviews	Students' perceptions about themselves as writers	Writer's workshop	Students' participation in writing workshops seemed to influence their writer identities.
Sessions et al. (2016)	USA	5th	30	Qualitative	Interviews	Attitude	Story writing using picture books as model text	Both students receiving traditional instruction and students working with iPad apps increased their writing skills. However, students using iPad apps produced better texts, experienced the writing activity as more socially engaging, and showed higher levels of motivation to write.

Study	*Country*	*Grade level(s)*	*Student sample size*	*Methodology*	*Type of student self-report(s)*	*Motivation construct*	*Type of writing task*	*Main findings*
Silver & Lee (2007)	Singapore	4th	33	Mixed methods	Survey including two open-ended questions	Attitude	Two 120-word narrative compositions (with the topics 'A robery in the park' and 'An outing to the kelong'. Each topic had three pictures to guide students in their writing, but they were required to come up with their own conclusions)	Teacher feedback characterized by advice encouraged the most revision. However, most of the revisions were judged to be minimal, and only 10% of the revisions were considered successful. Students preferred praise and advice, but although they disliked criticism, this feedback type also led to revisions.
Snyders (2014)	USA	Kindergarten	3	Qualitative	Interviews and videotaped student-teacher conferences	Self-efficacy<br>Writer identity	Book writing during workshop	Prior writing experiences and participation in writing workshops where students could associate themselves with the authors explored during mini-lessons influenced the students' identities as writers.
Teague et al. (2010)	Mexico	1s and 4th	6	Qualitative	Interviews	Attitude	Various writing tasks (authors noticed many copious school writing including dictations, copying, and simple texts)	Results indicated that home and community texts were seldom included in classroom instruction, and that writing attitudes were often associated with expectations of correctness and neatness.
Truax (2018)	USA	2nd and 3rd	56	Mixed methods	Interviews, survey, students' feedback on exit slips, and recorded teacher-student conferences	Growth mindset<br>Self -concept<br>Value	Writing workshop	Although quantitative results did not show significant effects, qualitative findings indicated that growth mindset feedback combined with objective compliments increased students' writing motivation.

Study	*Country*	*Grade level(s)*	*Student sample size*	*Methodology*	*Type of student self-report(s)*	*Motivation construct*	*Type of writing task*	*Main findings*
Tunks (2010)	USA	5th	215	Quantitative	Survey	Attitude	Descriptive writing<br>Narrative writing<br>Expository writing	Students’ motivation to write decreased after preparing for and taking the state-mandated exam.
Unal (2010)	Turkey	4th and 5th	2315	Quantitative	Survey	Writing disposition (divided into three subcategories: confidence, persistence, and passion)	N/A	Results indicated that students’ writing disposition levels are positive, but that the writing disposition levels among schools vary.
Wilson & Trainin (2007)	USA	1st	198	Quantitative	Survey	Attributions<br>Perceived competence<br>Self-efficacy	Survey questions about story writing	Results suggested that students can differentiate their self-efficacy for reading, writing, and spelling and that literacy attributions mediate between achievement and self-efficacy.
Zumbrunn et al. (2017)	USA	5th	114	Qualitative	Drawings of a recent writing experience and a written description of the drawings	Perception of themselves as writers<br>Beliefs about writing	N/A	Results indicated that students expressed both positive (e.g., joy) and negative (e.g., apathy, anxiety) emotions in relation to writing. Often, drawings illustrating positive experiences depicted teachers near the students, whereas drawings illustrating negative experiences depicted the teacher further away. Students also expressed feeling more motivated to write when confident in their writing abilities and allowed choice opportunities.

Study	*Country*	*Grade level(s)*	*Student sample size*	*Methodology*	*Type of student self-report(s)*	*Motivation construct*	*Type of writing task*	*Main findings*
Zumbrunn et al. (2019)	USA	5th	263	Mixed methods	Survey including two open-ended questions	Students’ writing enjoyment and aversion<br>Students’ perceptions of teacher writing enjoyment	Survey questions about writing in general	Quantitative findings indicated a positive relationship between students’ perceptions of teacher writing enjoyment and their own writing enjoyment. Qualitative findings suggested that their writing enjoyment and aversion is influenced by their writing preferences, as well as their mood and motivation.
